# Parental experiences and breastfeeding outcomes of early support to new parents from family health care centres—a mixed method study

**DOI:** 10.1186/s12884-022-04469-6

**Published:** 2022-02-23

**Authors:** Sandra Saade, Renée Flacking, Jenny Ericson

**Affiliations:** 1 School of Health and Wellfare, Dalarna University, Högskolegatan 2, 791 31 Falun, Sweden; 2 Centre for Clinical Research Dalarna, Uppsala University, Falun, Sweden; 3 Department of Paediatrics, Falu Hospital, Falun, Sweden

## Abstract

**Background:**

Early parenthood is a sensitive period for parents. Parents may feel uncertain about their new roles and unsure about where to find trusted information and support. The aim of this study was to explore the association between breastfeeding and early home visits and a proactive telephone support intervention and to describe parental experiences.

**Method:**

This study was conducted as a mixed method study with a convergent design using qualitative data from the written comments of parents, and the quantitative data consisted of demographics, breastfeeding, and Likert questions about parents’ satisfaction with the early home visit and telephone support. Historic control (2017–2018) and intervention (2019–2020) data were collected from one family health care centre, and control (2019–2020) data were collected from another family health care centre.

**Results:**

In total, 838 infants, 42 mothers and 38 fathers contributed to the data in the study. The intervention group had a statistically significantly earlier home visit than the control groups. Early home visits and proactive telephone support to parents with newborn infants were not associated with breastfeeding outcomes up to six months after birth, but we could not exclude the possibility that this was a consequence of our observational study design. However, the early home visit was appreciated by the parents where they received both practical and emotional support.

**Conclusions:**

Although the intervention was not associated with breastfeeding, the parents appreciated the service. This shows the importance of continuing to investigate how and which support parents of newborn infants need and the effects of such support, including interventions to provide optimal support to facilitate continued breastfeeding.

**Supplementary Information:**

The online version contains supplementary material available at 10.1186/s12884-022-04469-6.

## Background

The first weeks after the infant is born constitute a vulnerable period for the new family, as parents may feel uncertain in their parenting roles and are unsure about where to find trusted information [1]. Both mothers and fathers may feel stressed early on [2]. Support from health care professionals may be important for parents to find their parental roles and for breastfeeding [3–5].

The best food for the infant is breast milk. The recommendations are to initiate breastfeeding within the first hour of life and continue to exclusively breastfeed during the first six months of life [6]. Breastfeeding promotes health for both infants and mothers, and there are both short-term and long-term effects, such as decreased mortality and morbidity rates [7]. Worldwide, approximately 44% of infants between the ages of 0 and 6 months are exclusively breastfed [8]. In Sweden, the incidence of breastfeeding is decreasing. The rates of exclusively breastfed infants at one week of age decreased from 89% in 2004 to 75% in 2017 and from 19 to 13% at 6 months [9].

Mothers have various wishes regarding breastfeeding support. Mothers need to feel safe, and they want consistent advice about breastfeeding with practical support, in which positive attitudes towards breastfeeding from midwives are important. A comfortable environment is facilitative, and home visits are appreciated by mothers [10]. Mothers have reported that informal face-to-face support and their own determination helped them continue breastfeeding after six months [11]. A study conducted on mothers with preterm infants showed that proactive support (initiated by the health professional) led to more positive experiences than reactive support (initiated by the mother). Feelings of satisfaction, more involvement and empowerment were identified [12]. However, proactive telephone support did not affect breastfeeding in this population [13]. Nevertheless, proactive breastfeeding peer support through telephone has had a positive impact on breastfeeding in other studies [14, 15].

Three periods are identified as important for mothers to continue breastfeeding: breastfeeding support before birth, around the time of birth and in the early weeks after birth [16]. It is known that more than 50% of mothers cease breastfeeding during the first five weeks after birth, which makes this early period important for maintaining breastfeeding [17]. For many mothers, there is a gap in the health care system after the mother and infant have left the maternity unit and where the child health service takes over the responsibility. It may be unclear to mothers where to turn if problems with breastfeeding occur or support is needed [18, 19]. Since it is unclear how support should be provided to parents in the first days after birth, we hypothesized that an early home visit and proactive telephone support would capture potential breastfeeding difficulties and provide support at an early stage, which could facilitate continued breastfeeding. Thus, we conducted a study with the aim of exploring the association between breastfeeding and an early home visit and proactive telephone support intervention as well as to describe parental experiences.

## Method

### Design

This study was conducted as a mixed method study with a convergent design according to Creswell (2017) [20]. In this study, qualitative and quantitative data were collected during the same period. Quantitative and qualitative data were analysed separately and brought together to obtain a better understanding of parents’ experiences of early support and its association with breastfeeding.

### Setting

In Sweden, the mean time for staying in the hospital after an uncomplicated vaginal birth is 12–24 h, and the shortest time before discharge is 6 h. In the first week after birth, the hospital and maternity ward are responsible for the care of the infant and mother. After the first week, child health services are responsible for the infant’s health [19]. Child health services are an optional offered service free of charge to all children aged 0–5 years, and it is estimated that participation is almost 100%. The National Board of Health and Welfare sets the guidelines for the child health service program in the country. There are interventions offered to all children that aim to promote health and prevent any kind of problem, whether physical, psychological, or social. Special interventions are also offered to children depending on the individual child and family needs in collaboration with, for example, other health care providers, social workers, or other resources [21]. Each child health service centre provides care by physicians and nurses, and the nurses have a specialist degree in either primary or paediatric care. When needed, the nurses refer to other professions, such as psychologists or speech and language therapists. In some places, child health services are included in family health care centres. A family health care centre should include maternity and child health care, an open preschool and preventive social service, which leads to many professionals under the same roof [22]. There is a recommendation that families with newborn infants have two visits to the child health service/family health care centre from day seven to the third week after birth, of which one is a home visit within two weeks after discharge [21]. The present study took place in one family health care centre with six health care nurses and approximately 280 newborn infants per year in a medium-sized municipality with 55,000 citizens located in the middle of Sweden. The other family health care centre with three health care nurses and approximately 140 newborn infants per year in the same municipality was used as a control group. Both centres had both wealthy and vulnerable residential areas including both high and low socioeconomic status families to care for.

### Intervention

In November 2018, the intervention family health care centre started a project where they offered an early home visit aiming at day three after birth and thereafter daily proactive phone calls, i.e., one of the family health care nurses called the family until the family had their first appointment at the family centre. The early home visit was intended to support breastfeeding and parents’ well-being early after discharge from the maternity/delivery unit compared to usual care. However, there were no instructions on how the early visits should be carried out, and it was up to the individual nurse to structure the visits. Usual care, i.e., a home visit or visit at the family centre, was performed later (within two weeks), where for example, infant growth, life situation, health and safety were addressed. The intervention was offered to all families who had a newborn infant, and the families could decline home visits if they did not want one without further explanation. The other family health care centre was used as a control and continued with usual care, i.e., home visits within the first two weeks after birth.

### Data collection

The present study consists of two parts and includes both qualitative and quantitative data.

**Fig. 1:**
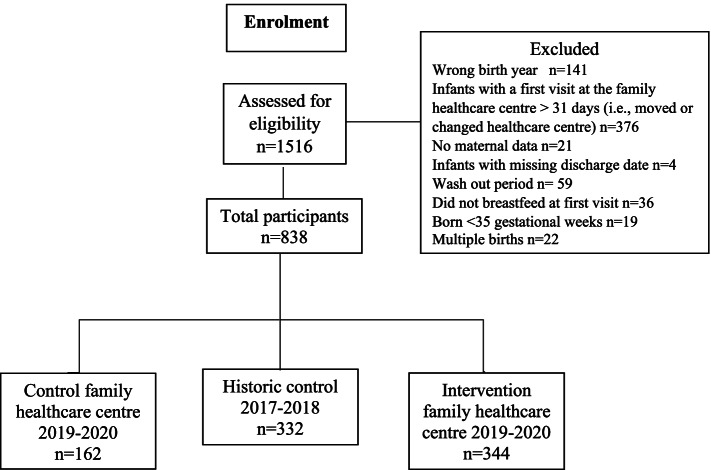
Flowchart of the inclusion and exclusion of infants in Part 1 of the study

Part 1 consists of deidentified parts of the records from all newborn infants who were registered at the family health care centres. The data were extracted from two different journal systems: one for the mother (Obstretrix®, Siemens AG, journal system for pregnancies and deliveries) and one for the infant (Take Care®, CompuGroup Medical, journal system for health and hospital care). The Region Dalarna provided the extraction of the data. At the intervention family health care centre, historic control data were collected between 2017–01–01 and 2018–09–31, and intervention data were collected between 2019–01–01 and 2020–06–01. Data from the control family health care centre were collected between 2019–01–01 and 2020–06–01. During autumn 2018, the nurses at the family health care centre received an education and started to plan for the intervention together with the author JE. The nurses could have started the earlier home visits before the study started. Therefore, a wash out period was set between 2018–09–31–2018–12–31 in the historic control group to be sure that this was not the case. In total, 838 infants contributed to the data in this part. A flowchart of the included and excluded infants (with reasons) is shown in Fig. 1. The collected demographic data were the mothers’ age, cohabiting status (single/other living situation or cohabiting), parity (primipara or multipara), pregnancy length (weeks), mode of delivery (vaginal or caesarean section), infant sex (female/male), and hospital discharge day. The collected outcome variables were the first visit day from the family health care nurse, breastfeeding (exclusive, partial, no) at discharge from maternity hospital and at every visit at the family centre up to six months after birth. Breastfeeding in this study was defined according to the WHO definition of exclusive, partial and no breastfeeding [6].

The second part of the study consisted of data collected from both parents during pregnancy and two months after birth via questionnaires. The parents were asked to participate in the study during their visit at the midwife at the intervention family health care centre around pregnancy week 28. Approximately 150 parents were asked about participation in the study during 2019. The parents who consented to participate in the study answered two questionnaires, one before childbirth, which contained demographic data and questions about the parent’s expectations for parenthood, breastfeeding, and sleep. Two months after childbirth, the parents received a second questionnaire where they answered questions about, for example, the birth and infant demographics, breastfeeding and their experiences of the early home visit and telephone support. Only the demographic data and questions of the early home visit and telephone support were used in this study. The following questions were asked in the second questionnaire: “*The early home visit from the family health care centre did suit us well*” and “*The telephone support provided after the early home visit did suit us well*”, with a five-point Likert scale from “totally agree” to “do not agree at all”. There was also one open-ended question: “*Feel free to describe your experiences around the early visit from the family centre and telephone support (what was good, what was not good, what did you want?)*”. In total, 42 mothers and 38 fathers participated in this part of the data collection.

### Data Analysis

The quantitative data were analysed using IBM SPSS Statistics for Windows, Version 27.0. Armonk, NY: IBM Corp and R (R Core Team, 2021) version 4.1.0 with the add-on package icenReg. Descriptive data were analysed and presented using numbers, percentages, means, standard deviations (SD), medians and interquartile ranges (IQRs) depending on the data distributions. To assess possible associations between the three groups (intervention, historic control, and control) and breastfeeding (any vs. no breastfeeding) in Part 1, a survival analysis was used. The R package icenReg was used for the analysis, which is a method for analysing cox regression models for interval censored data. Interval censored data occur when an event time is known only up to an interval. In this study, breastfeeding was registered at each visit; thus, the exact date of changes in breastfeeding was not known. The Cox regression model was adjusted for maternal age, parity, delivery mode, and hospital discharge day.

The Likert scale questions in Part 2 about how the early home visit and the telephone support suited the parents were analysed separately for mothers and fathers with descriptive statistics and then compared using an independent t-test.

The qualitative data in Part 2, i.e., the written comments from the open-ended questions in the questionnaire two months after birth, were analysed with an inductive content analysis inspired by Elo and Kygnas (2008) [23]. The authors began with reading all the written comments. After reading the written comments many times and understanding the whole, the authors started writing notes next to every comment. These notes were then written in a separate document, and subcategories were formulated by grouping similar notes together. Thereafter, the subcategories were grouped into categories. After this step, the authors compared the analysis together, and after discussions, a result was created. The mothers’ and fathers’ written comments were first analysed separately and then compared. To strengthen the results, quotes from the written comments were used. Each quote was marked with the parents’ specific code to preserve confidentiality and shows whether it was written by a mother or a father.

### Ethical considerations

The study received ethical approval from the Swedish ethical review authority Dnr: 2019–00,294. The Region of Dalarna has given approval for accessing health records and extracting the deidentified data.

## Results

The participants’ demographic information in Part 1 is presented in Table 1.

**Table 1:** Characteristics of participating infants (*N* = 838) in Part 1 and mothers (*N* = 42) and fathers (*N* = 38) in Part 2

	Part 1			Part 2	
	Control	Historic control	Intervention	Mothers	Fathers
**Demographic variables**	n (%) median [IQR] mean ± SD			n (%) median [IQR] mean ± SD	
Numbers	*n* = 162	*n* = 332	*n* = 344	*n* = 42	*n* = 38
Age, years	30.5 ± 5.5	30.7 ± 5.4	31 ± 5.2	32 ± 4.9	34 ± 6.9
Educational level				*n* = 42	*n* = 36
Higher education				31 (74)	17 (47)
Upper secondary school				9 (21)	19 (53)
Compulsory school				2 (4.8)	0
Work	*n* = 85	*n* = 172	*n* = 184		
Unemployed	5 (5.9)	34 (20)	30 (16)		
Part time	45 (53)	71 (41)	76 (41)		
Full time	35 (41)	67 (39)	78 (42)		
Cohabitation status	*n* = 161	*n* = 317	*n* = 327		
Cohabitation	148 (93.1)	293 (92.4)	296 (90.5)		
Single/another living situation	11 (6.9)	24 (7.6)	31 (9.5)		
Birth country					
Sweden				39 (92.9)	36 (94.7)
Other				3 (7.1)	2 (5.3)
Parity	*n* = 160	*n* = 324	*n* = 336	*n* = 42	*n* = 37
Primipara	60 (38)	129 (40)	125 (37)	20 (48)	18 (49)
Multipara	100 (62)	195 (60)	211 (63)	22 (52)	19 (51)
Delivery mode	*n* = 160	*n* = 329	*n* = 337	*n* = 42	*n* = 38
Vaginal birth	138 (85)	279 (84)	297 (88)	32 (76)	29 (76)
C-section	22 (14)	45 (14)	40 (12)	10 (24)	9 (24)
**Infant variables**					
Gestational age at birth, weeks	39 [2]	39 [2]	39 [2]	39 [2]	39 (2.25]
Sex					
Female	74 (46)	155 (47)	169 (49)	26 (62)	22 (58)
Male	88 (54)	177 (53)	175 (51)	16 (38)	16 (42)
Length of hospital stay, days	2 [2]	2 [2]	2 [2]		
Breastfeeding at discharge from maternity unit	*n* = 156	*n* = 319	*n* = 329		
Exclusive	141 (90.4)	297 (93.1)	309 (89.8)		
Partial	13 (8.3)	19 (6)	20 (6.1)		
No	2 (1.3)	3 (0.9)	0		

*SD* standard deviation, *IQR* interquartile range

### Part 1: Day of early home visit and breastfeeding outcomes

**Fig. 2:**
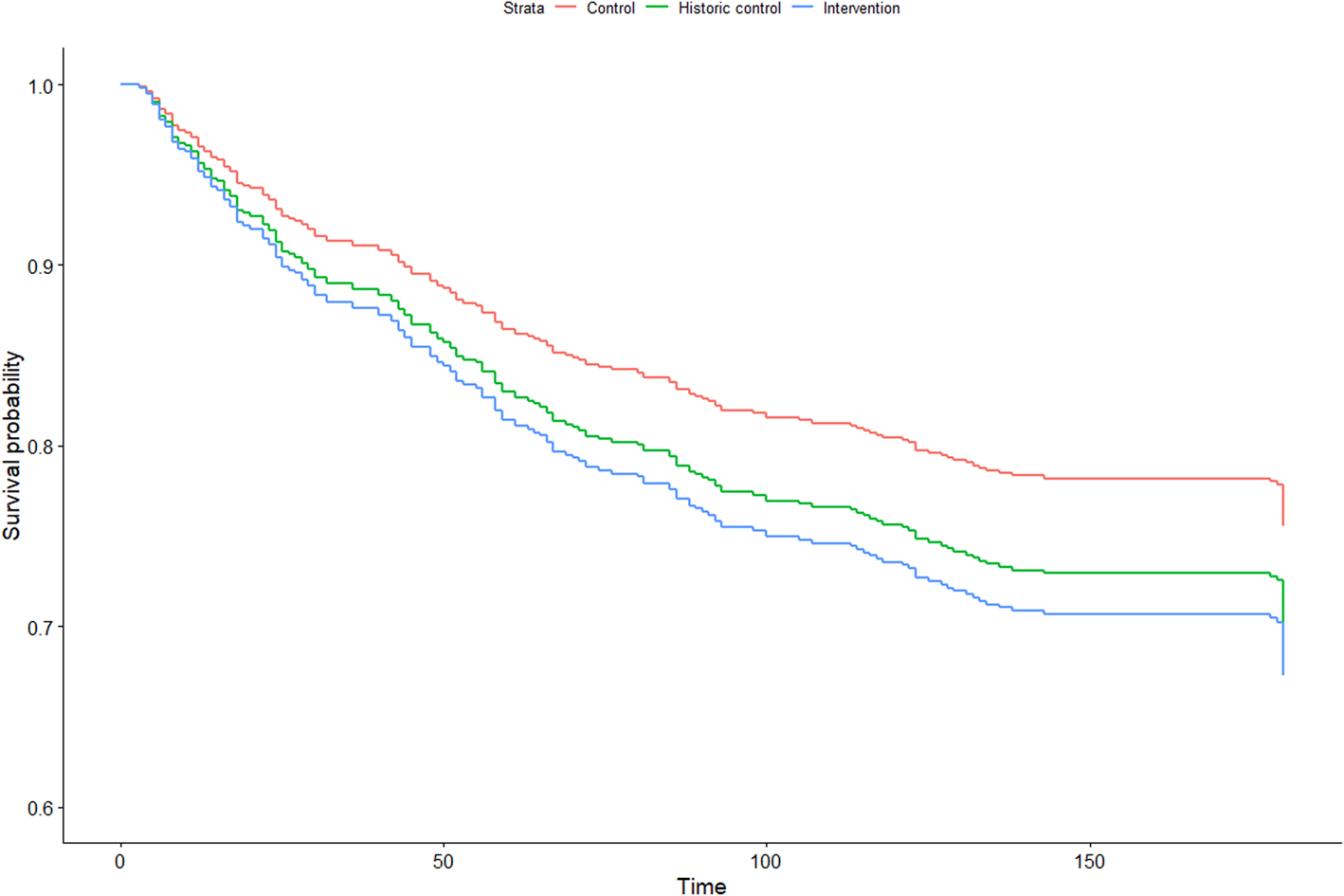
Survival curve over cessation of breastfeeding in the three groups (control, historic control, and intervention) during six months after birth (i.e., 180 days) in Part 1

The results showed that the median first visit day from the family health care nurse to the family was 6 days (IQR 4) in the intervention group and 8 (IQR 4) in the historical control and control groups (*p* < 0.001). The median number of days with exclusive breastfeeding up to 6 months was 112.5 [IQR 85.5] in the control group, 115 [IQR 106.5] in the historical control group and 95 [IQR 103.5] in the intervention group. There was no statistically significant difference in breastfeeding between the three groups in the Cox regression model (Fig. 2 and Table 2). The only statistically significant factor for breastfeeding in the model was maternal age, where older mothers breastfed longer (Table 2). When analysing exclusive breastfeeding compared to any/no breastfeeding, there were no statistically significant differences between the three groups, and the results are not presented.

**Table 2:** Results from the Cox regression model of associations between the three groups (intervention, *n* = 344, historic control, *n* = 332 and control, *n* = 162) and breastfeeding (any vs. no breastfeeding) in Part 1 presented with Hazard Ratio (HR), 95% confidence interval (CI) and p value

	HR (95% CI)	*p* value
Control group	ref	
Historic control group	1.26 (0.80–1.98)	0.31
Intervention group	1.44 (0.92–2.24)	0.11
Length of hospital stay, days	1.04 (0.95–1.13)	0.36
Maternal age, years	0.94 (0.91–0.97)	< 0.001
Vaginal birth	0.64 (0.39–1.03)	0.06
Primipara	1.08 (0.75–1.53)	0.69

### Part 2: Parental experiences of the support

The result of the Likert questions about whether the early home visit and telephone support suited the parents showed that the mean for the early home visit for the mothers was 4.12 (SD 1.50) and for the fathers 4.14 (SD 1.49). For the daily phone calls, the mean was 3.56 (SD 1.50) for the mothers and 3.46 (SD 1.44) for the fathers (Table 3).

**Table 3:** Results of the five-point Likert questions about if the early home visit and telephone support suited the parents in Part 2. Presented with mean, standard deviation (SD) and p value

	Mothers	Fathers	
	mean ± SD		*p* value
	*n* = 41	*n* = 37	
*"The early home visit from the family centre did suit us well"*	4.12 ± 1.50	4.14 ± 1.49	0.97
	*n* = 41	*n* = 35	
"*The telephone support provided after the early home visit did suit us well"*	3.56 ± 1.50	3.46 ± 1.44	0.76

Four dyads did not receive any early home visit, and another four dyads did not have early phone calls. A reason for not having any early home visit was a long stay at the hospital, which made it impossible to have an early home visit and telephone support. There were also parents who did not receive an early home visit and early telephone support for unknown reasons. However, these families did get the usual support.

**Fig. 3:**
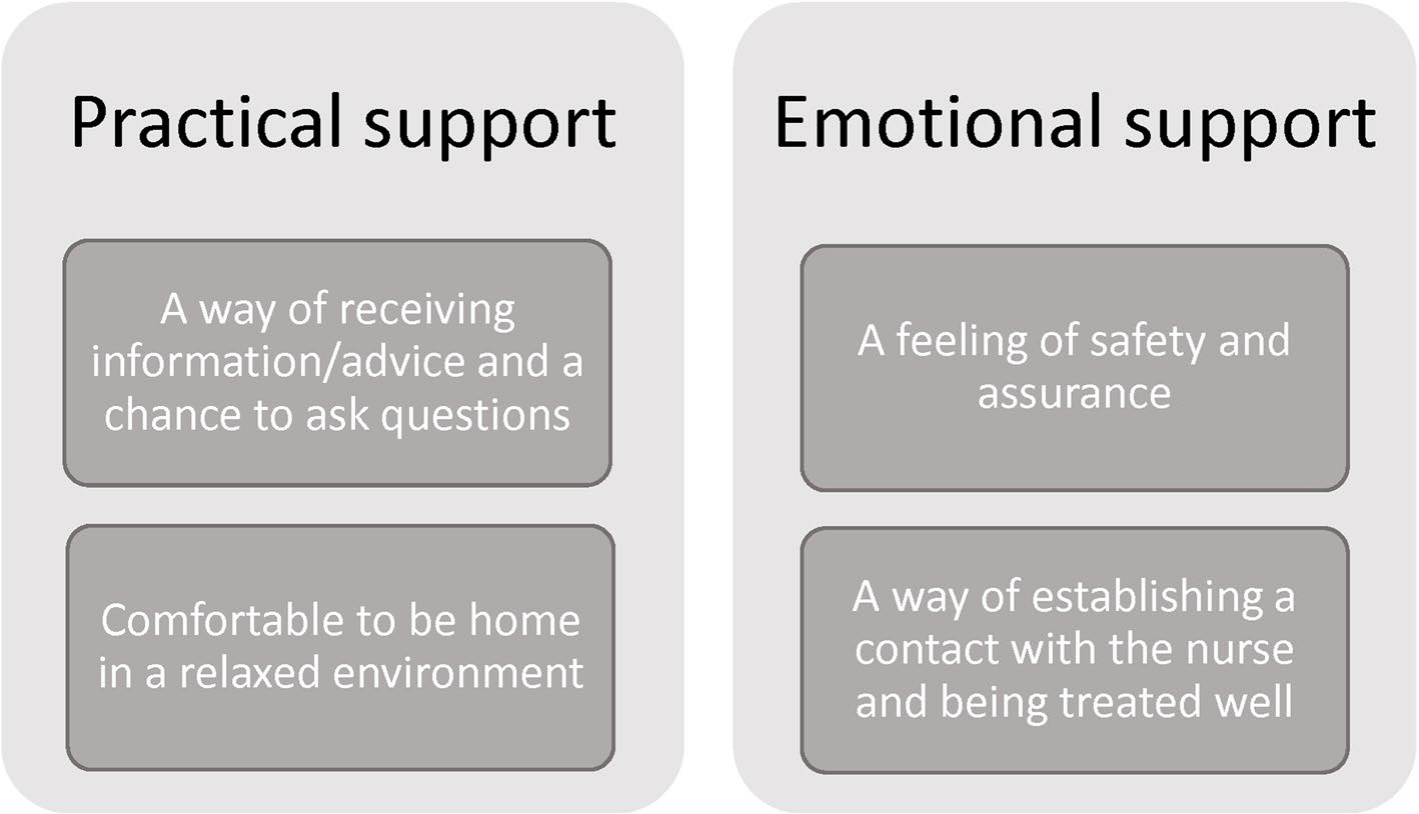
Subcategories and categories about the parents’ experiences of the early home visit and telephone support in Part 2

In total, 30 mothers and 18 fathers left written comments on the open-ended question about early support. The analysis of the open-ended question resulted in four subcategories and two categories. The four subcategories were “*A way of receiving information/advice and a chance to ask questions”, “Comfortable to be home in a relaxed environment”, “A way of establishing a contact with the nurse and being treated well”* and *“A feeling of safety and assurance”*. The categories were “*Practical support”* and “*Emotional support,* Fig. 3.

#### Practical support

In the written comments, the parents described the support as a way of getting practical help. It could be about breastfeeding, what to do in certain situations, or confirmation that things are done the right way. Many parents saw the home visit as a comfortable way to get help without leaving the home with a newborn. In the category “*A way of receiving information/advice and a chance to ask questions”,* the mothers and fathers valued the opportunity to ask questions and to receive information. The parents did see the home visit as an opportunity to ask the questions they had and obtain general information about what was coming next and specific information about the infant.

“*Good with all information about vaccinations, phone numbers and where to turn”,* (mother, code 98), “…*The home visit was good for ask all questions and share thoughts”,* (father, code 114).

Breastfeeding support was mentioned almost only by the mothers and in terms of practical support. The mothers appreciated early support and advice on breastfeeding and that the nurses could observe and show them how to do during the home visit.

*“It was at a good time, although we had only been home 1 day. Great to get help/advice with breastfeeding in relaxed environment.”* (Mother, code 120)

Some mothers asked for more support on breastfeeding and wanted the nurse to observe breastfeeding. Some mothers expressed a lack of support with breastfeeding or that they did not receive the right advice and help that they needed.

“*It was nice to get a home visit and not have to leave the home. However, the visit ended in many tears. Difficult advice regarding breastfeeding made it not work. Finally, I got more relaxing advice from the maternity unit. The advice from the child health services was to breastfeed every two hours and cup feeding without instructions on how milk expression was done. This created despair and a sense of failure when it did not work”* (mother, code 16).

The only father who mentioned breastfeeding was the father with the same code (code 16). This father expressed that advice on breastfeeding was not well considered and created stress and anxiety in the mother. Another subcategory was *“comfortable being home in a relaxed environment”*. In this subcategory, the parents wrote comments that showed that it was more comfortable and easier to stay at home with an infant instead of leaving the home. The mothers also expressed that the environment at home was more relaxed and that the early home visit was appreciated because of the opportunity to avoid being around many people.

*“…it was more relaxing to be at home”,* (mother, code 48), “*Feels safe and easy not to have to leave with the child”,* (father, code 4).

#### Emotional support

In the other category*, emotional support,* the parents expressed more feelings and that the support contained emotional support.

In the subcategory “*A feeling of safety and assurance”,* the parents expressed positive feelings about the early home visit and telephone support, and words such as assurance, safety, and nice were used. Thus, the support gave them a sense of security:

“…*It felt safe and personal*”, (mother, code 65), *“It felt safe, and that I was not alone”*, (father code, 89).

In the subcategory “*A way of establishing contact with the nurse and being treated well”*, the parents described the home visit as a way of getting to know the nurse and establishing personal contact before a visit to the family health care centre. They also wrote comments on how the nurse was during the home visit and the nurses’ qualities. The parents seemed to appreciate when the nurse listened to their needs and requests.

” *I had a good contact with the nurse, and she listened to my needs/wishes for example about visiting hours/visits at the family center” (mother code 55). “Nice, understanding, accommodating…”, (father, code 89).*

## Discussion

In this study, an early home visit was not associated with breastfeeding up to six months after birth. However, the parents who received an early home visit appreciated the service, which was described as providing both practical and emotional support. The lack of association between the early home visit and breastfeeding may be due to a ceiling effect. Sweden has a high initiation rate of breastfeeding; in this study, it was > 90% breastfeeding at discharge from the maternity unit, which is consistent with national numbers [9]. It may be difficult to affect breastfeeding when most mothers already breastfeed, and it becomes a small group to influence, which makes it difficult to find effects. However, the intention of the intervention was to facilitate mothers to continue breastfeeding and prevent breastfeeding problems, as breastfeeding decreases quite rapidly in the first weeks after birth. Second, the breastfeeding support that was offered may not have been optimal in terms of the family health care nurse’s ability to base the support on the individual mother’s situation, which was shown in some of the mothers’ written comments. The nurses performing the intervention did not have any special breastfeeding education before the start of the intervention. Some nurses were specialists in paediatric nursing, several had worked as neonatal nurses and had extensive experience in breastfeeding support, and some were specialists in primary care nursing. Although the paediatric and district nurses were equally distributed between the two family health care centres, it is possible that a few more unexperienced nurses worked at the intervention family health care centre, however it is unclear how this have affected the result in the study. This means that the nurses may have had various knowledge about breastfeeding and support. Earlier research about breastfeeding support offered to mothers showed that the quality of the support differed depending on the individual health professional who provided support in terms of their knowledge and support style [24]. Third, the breastfeeding prevalence in Sweden is currently declining by approximately one percent per year [9]. Thus, this probably explains the higher breastfeeding in the historic control group. In conclusion, it seems that it is not enough to offer only an early home visit to affect breastfeeding prevalence. Supportive interventions, training efforts for health professionals and the Baby Friendly Hospital Initiative [25] have been proven to increase breastfeeding to some extent in earlier studies [26, 27]. Training efforts provided to family health care nurses may be needed to increase their ability to provide breastfeeding support based on the individual mother's conditions and needs.

The parents were overall satisfied with the early home visit and telephone support and described that they experienced both practical and emotional support. This is positive and shows that it is important for child health nurses to visit the family as early as possible after discharge from the hospital. Although the intervention did not have an association with breastfeeding, it could have been associated with other important areas, such as parental stress and feelings of competence or well-being, which should be investigated in further studies.

One reflection was that many comments were about breastfeeding support, although the question was not asked specifically about breastfeeding, which shows that breastfeeding is an important area for new parents and that health care providers need to take that into account in early support after birth. However, the written comments on breastfeeding only contained comments of practical support, such as showing the mother, providing advice or observing a breastfeed. Emotional aspects of breastfeeding support have been described as important by mothers in earlier research [24, 28]. Perhaps the mothers focus on early breastfeeding in practical terms, and emotional aspects emerge later.

### Limitations

This study has several limitations. The main threat in part 1 is systematic differences in the characteristics of the different groups due to the non-randomized observational design. This affects the internal validity of this study, which could explain the negative tendency of the intervention in this study. We assumed that the groups, when adjusting for available confounders, were equivalent i.e., the three groups did not differ in the characteristics we had access to, in our interpretation of the intervention effect. However, there is a potential risk that the groups are still not completely comparable as the control group's family health care centre was considerably smaller. For the historical control group, there could be circumstances that have changed in the society influencing breastfeeding that we could not control for. Furthermore, we could not adjust for other potential confounders associated with breastfeeding because of lack of data in Part 1, such as maternal educational level, maternal and infant health, pregnancy data and breastfeeding satisfaction. In addition, there were limitations in the extraction of data, where the various journal systems were not synchronized and we lacked data on eventual readmissions/admissions to the neonatal/paediatric unit, which makes the time to the first home visit uncertain. The results should be interpreted with that in mind. However, the data were collected in the same way in all three groups. Other limitations were, we only had follow-up data on breastfeeding in Part 1. Thus, it was not possible to investigate possible associations with other important outcomes, such as parental stress, competence, or well-being. Furthermore, the depth of the described experiences of the early home visit and telephone support in Part 2 are lacking when the comments were relatively short. A more in-depth investigation of the parental experience of early home visits and usual care, for example, using an interview study design, may provide a deeper understanding. Both are important to investigate in further studies. In Part 2, the small number of participating parents, mostly Swedish, highly educated, and breastfeeding mothers and their partners, may have biased the results.

## Conclusion

An early home visit to parents with newborn infants was not associated with breastfeeding outcomes up to 6 months after birth. However, the early home visit was appreciated by the parents when they received both practical and emotional support. This shows the importance of continuing to investigate how and which support parents of newborn infants are in need for and the effects of such support, including interventions to provide optimal support to facilitate continued breastfeeding.

## Supplementary Information

**Additional file 1. **Areas of aspects and questions from parents.

## Data Availability

The datasets generated and analysed during the current study are not publicly available due for ethical and legal reasons but are available from the corresponding author on reasonable request.
