# Supplementary material for: Parental experiences and breastfeeding outcomes of early support to new parents from family health care centres—a mixed method study
Source: BMC Pregnancy Childbirth. 2022 Feb 23;22:150. doi: 10.1186/s12884-022-04469-6 (PMC8867764; doi:10.1186/s12884-022-04469-6)
Supplement: Supplementary file 1 — Additional file 1. Areas of aspects and questions from parents. [file 12884_2022_4469_MOESM1_ESM.docx]

**Supplementary file**

**Areas of aspects and questions from parents**

Answers to practical questions

- About vaccines
- Telephone numbers
- Where to turn
- Organization of the child health care service.

Home visit and telephone support

- Good with early support
- Convenient with a visit to the home
- Good not to have to get out so early
- Gave security to be in a home environment
- Good way to get in touch with your child health care nurse
- Nice to receive a call when feeling a bit low
- Did not need the telephone support

Breastfeeding advice/support

- Wanted more focus on breastfeeding
- Wanted to talk early about breastfeeding
- Wanted to be shown how to breastfeed
- Get individual adapted advice
- Wanted more advice/support
- Wanted good and suitable advice
- "Wrong" advice caused stress and anxiety
- There was a dissatisfaction with incorrect breastfeeding support which created a negative experience

Infant weight issues

- Good to be able to weigh the infant
